# Supplementary material for: Hair follicle epidermal stem cells define a niche for tactile sensation
Source: eLife. 2018 Oct 25;7:e38883. doi: 10.7554/eLife.38883 (PMC6226291; doi:10.7554/eLife.38883)
Supplement: Figure 1—source data 2. [file elife-38883-fig1-data2.docx]

**Figure 1–source data 2. Normalized Enrichment Score (NES) of Gene Set Enrichment Analysis (GSEA)**

| **Cdh3** | **CD34** | **Lgr6** | **Gli1** | **GO terms** |
| --- | --- | --- | --- | --- |
| 1.674 | 0.916 | -0.868 | 1.210 | NEGATIVE_REGULATION_OF_TRANSCRIPTION_DNA_DEPENDENT |
| 1.662 | 0.899 | -0.859 | 1.204 | REGULATION_OF_TRANSCRIPTION_FROM_RNA_POLYMERASE_II_PROMOTER |
| 1.655 | 0.930 | -0.808 | 1.201 | POSITIVE_REGULATION_OF_NUCLEOBASENUCLEOSIDENUCLEOTIDE_AND_NUCLEIC_ACID_METABOLIC_PROCESS |
| 1.639 | 0.907 | -0.863 | 1.186 | NEGATIVE_REGULATION_OF_RNA_METABOLIC_PROCESS |
| 1.571 | 0.981 | -0.898 | 1.228 | NEGATIVE_REGULATION_OF_NUCLEOBASENUCLEOSIDENUCLEOTIDE_AND_NUCLEIC_ACID_METABOLIC_PROCESS |
| 1.591 | 0.989 | -0.862 | 1.167 | POSITIVE_REGULATION_OF_TRANSCRIPTION |
| 1.561 | 0.928 | -0.817 | 1.148 | POSITIVE_REGULATION_OF_RNA_METABOLIC_PROCESS |
| 1.552 | 0.928 | -0.823 | 1.145 | POSITIVE_REGULATION_OF_TRANSCRIPTIONDNA_DEPENDENT |
| 1.492 | 0.990 | -0.850 | 1.252 | NEGATIVE_REGULATION_OF_METABOLIC_PROCESS |
| 1.474 | 1.000 | -0.854 | 1.249 | NEGATIVE_REGULATION_OF_CELLULAR_METABOLIC_PROCESS |
| 1.507 | 0.921 | 0.860 | 1.260 | NEGATIVE_REGULATION_OF_TRANSCRIPTION_FROM_RNA_POLYMERASE_II_PROMOTER |
| 1.508 | 1.006 | 0.897 | 1.099 | TRANSMEMBRANE_RECEPTOR_PROTEIN_TYROSINE_KINASE_SIGNALING_PATHWAY |
| 1.688 | -0.999 | -0.902 | 1.191 | REGULATION_OF_RNA_METABOLIC_PROCESS |
| 1.652 | -0.988 | -0.896 | 1.187 | REGULATION_OF_TRANSCRIPTIONDNA_DEPENDENT |
| 1.666 | -0.918 | -0.864 | 1.253 | REGULATION_OF_TRANSCRIPTION |
| 1.616 | -0.969 | -0.914 | 1.192 | REGULATION_OF_NUCLEOBASENUCLEOSIDENUCLEOTIDE_AND_NUCLEIC_ACID_METABOLIC_PROCESS |
| 1.510 | -0.938 | -0.885 | 1.134 | POSITIVE_REGULATION_OF_METABOLIC_PROCESS |
| 1.496 | -0.939 | -0.911 | 1.122 | POSITIVE_REGULATION_OF_CELLULAR_METABOLIC_PROCESS |
| 1.503 | -1.349 | -1.005 | 1.305 | MULTI_ORGANISM_PROCESS |
| 1.568 | -0.953 | 0.965 | 1.224 | TRANSCRIPTION_FROM_RNA_POLYMERASE_II_PROMOTER |
| 1.537 | -1.115 | 0.972 | 1.195 | PROTEIN_KINASE_CASCADE |
| 1.604 | -0.901 | 0.988 | 1.234 | PROTEIN_AMINO_ACID_PHOSPHORYLATION |
| 1.551 | -1.108 | 1.041 | 1.316 | REGULATION_OF_MAP_KINASE_ACTIVITY |
| 1.665 | -0.999 | 1.095 | 1.032 | FEEDING_BEHAVIOR |
| 1.629 | -0.837 | 1.165 | 1.424 | MAPKKK_CASCADE |
| 1.473 | -1.314 | 1.197 | 1.330 | RESPONSE_TO_BIOTIC_STIMULUS |
| 1.818 | 1.208 | -0.932 | 1.375 | MUSCLE_DEVELOPMENT |
| 1.715 | 1.316 | -1.093 | 1.341 | ENZYME_LINKED_RECEPTOR_PROTEIN_SIGNALING_PATHWAY |
| 1.544 | 1.286 | -1.377 | 1.332 | TRANSMEMBRANE_RECEPTOR_PROTEIN_SERINE_THREONINE_KINASE_SIGNALING_PATHWAY |
| 1.700 | 1.052 | 0.824 | 1.335 | NEGATIVE_REGULATION_OF_TRANSCRIPTION |
| 1.624 | 1.093 | 0.835 | 1.186 | POSITIVE_REGULATION_OF_TRANSCRIPTION_FROM_RNA_POLYMERASE_II_PROMOTER |
| 1.623 | 1.231 | -1.321 | 1.039 | FEMALE_PREGNANCY |
| 1.390 | -1.241 | -0.947 | 0.995 | REGULATION_OF_KINASE_ACTIVITY |
| 1.385 | -1.198 | -1.146 | 0.998 | G_PROTEIN_COUPLED_RECEPTOR_PROTEIN_SIGNALING_PATHWAY |
| 1.379 | -1.250 | -0.898 | 0.994 | REGULATION_OF_TRANSFERASE_ACTIVITY |
| 1.338 | -1.188 | -0.889 | 1.047 | REGULATION_OF_MOLECULAR_FUNCTION |
| 1.449 | -1.128 | -0.957 | 1.055 | REGULATION_OF_SIGNAL_TRANSDUCTION |
| 1.446 | -1.213 | -0.911 | 1.033 | REGULATION_OF_PROTEIN_KINASE_ACTIVITY |
| 1.301 | -1.199 | -0.998 | 1.278 | CELL_PROLIFERATION |
| 1.466 | -1.023 | 1.053 | 1.219 | PHOSPHORYLATION |
| 1.417 | -1.100 | 0.979 | 1.203 | POST_TRANSLATIONAL_PROTEIN_MODIFICATION |
| 1.425 | -0.968 | 1.021 | 1.091 | SECOND_MESSENGER_MEDIATED_SIGNALING |
| 1.275 | -1.170 | 1.081 | 1.087 | REGULATION_OF_DEVELOPMENTAL_PROCESS |
| 1.523 | -1.081 | -1.118 | 0.774 | NUCLEOCYTOPLASMIC_TRANSPORT |
| 1.516 | -1.095 | -1.097 | 0.759 | NUCLEAR_TRANSPORT |
| 1.555 | -1.082 | -1.215 | 0.785 | NUCLEAR_IMPORT |
| 1.629 | -1.056 | -1.234 | 0.804 | PROTEIN_IMPORT_INTO_NUCLEUS |
| 1.618 | -1.180 | 0.850 | 0.915 | B_CELL_ACTIVATION |
| 1.514 | 0.913 | -0.943 | 0.933 | STRIATED_MUSCLE_DEVELOPMENT |
| 1.213 | -1.495 | 1.241 | 1.433 | RESPONSE_TO_WOUNDING |
| 1.303 | -1.510 | 1.144 | 1.365 | RESPONSE_TO_EXTERNAL_STIMULUS |
| 1.304 | -1.822 | 1.296 | 1.300 | DEFENSE_RESPONSE |
| 1.384 | -1.278 | 1.255 | 1.621 | DETECTION_OF_EXTERNAL_STIMULUS |
| 1.211 | -1.096 | 1.146 | 1.599 | GROWTH |
| 1.139 | -1.151 | 0.978 | 1.487 | CATION_HOMEOSTASIS |
| 1.205 | 1.116 | 1.222 | 1.631 | NEURON_DIFFERENTIATION |
| 1.152 | 1.097 | 1.242 | 1.577 | NEURON_DEVELOPMENT |
| 1.159 | 1.075 | 1.114 | 1.537 | GENERATION_OF_NEURONS |
| 1.023 | 1.012 | 1.089 | 1.520 | NEUROGENESIS |
| 1.233 | 1.168 | 0.943 | 1.375 | NERVOUS_SYSTEM_DEVELOPMENT |
| 1.605 | 1.198 | 1.168 | 1.634 | GLUTAMATE_SIGNALING_PATHWAY |
| 1.461 | 1.090 | 1.112 | 1.654 | BRAIN_DEVELOPMENT |
| 1.450 | 1.273 | 0.969 | 1.585 | ORGAN_MORPHOGENESIS |
| 1.603 | 1.140 | 1.287 | 1.360 | CYCLIC_NUCLEOTIDE_MEDIATED_SIGNALING |
| 1.487 | 1.102 | 1.180 | 1.412 | CELL_CELL_SIGNALING |
| 1.624 | 1.024 | 1.160 | 1.300 | G_PROTEIN_SIGNALING_COUPLED_TO_CYCLIC_NUCLEOTIDE_SECOND_MESSENGER |
| 1.162 | -0.900 | 1.621 | 1.517 | SODIUM_ION_TRANSPORT |
| 1.081 | -1.806 | 1.593 | 1.514 | DEFENSE_RESPONSE_TO_BACTERIUM |
| 1.261 | -1.316 | 1.669 | 1.552 | METAL_ION_TRANSPORT |
| 1.251 | -1.175 | 1.430 | 1.604 | TISSUE_DEVELOPMENT |
| 1.083 | 1.052 | 1.476 | 1.761 | DEVELOPMENTAL_MATURATION |
| 1.055 | 1.061 | 1.603 | 1.492 | MONOVALENT_INORGANIC_CATION_TRANSPORT |
| 1.022 | -1.147 | 1.529 | 1.558 | ECTODERM_DEVELOPMENT |
| 0.957 | -1.205 | 1.505 | 1.554 | EPIDERMIS_DEVELOPMENT |
| -0.800 | -1.495 | 1.683 | 1.511 | KERATINOCYTE_DIFFERENTIATION |
| -0.693 | -0.899 | 1.608 | 1.414 | REGULATION_OF_DEFENSE_RESPONSE |
| -1.175 | -0.977 | 1.651 | 1.799 | SYNAPTOGENESIS |
| -1.526 | -1.289 | 1.591 | 1.748 | SYNAPSE_ORGANIZATION_AND_BIOGENESIS |
| -0.849 | 0.874 | 1.491 | 1.286 | ALCOHOL_METABOLIC_PROCESS |
| -1.015 | -1.208 | 1.454 | 1.344 | CATION_TRANSPORT |
| -1.170 | -1.286 | 1.437 | 1.190 | LIPID_METABOLIC_PROCESS |
| -1.047 | -1.143 | 1.345 | 1.164 | CELLULAR_LIPID_METABOLIC_PROCESS |
| -0.947 | -1.732 | 1.647 | 1.132 | INNATE_IMMUNE_RESPONSE |
| 1.501 | 1.102 | 1.530 | 1.662 | EXCRETION |
| 1.521 | -1.415 | 1.419 | 1.633 | DETECTION_OF_STIMULUS |
| 1.663 | -1.418 | 1.902 | 1.663 | CALCIUM_INDEPENDENT_CELL_CELL_ADHESION |
| 1.497 | 1.355 | 1.321 | 1.673 | CELL_MATURATION |
| 1.465 | 1.344 | 1.219 | 1.599 | ANATOMICAL_STRUCTURE_MORPHOGENESIS |
| 1.711 | 1.409 | 1.291 | 1.590 | PATTERN_SPECIFICATION_PROCESS |
| 1.388 | 1.539 | 1.376 | 1.665 | POTASSIUM_ION_TRANSPORT |
| 1.609 | 1.805 | 1.517 | 1.718 | AXON_GUIDANCE |
| 1.899 | 1.849 | -0.724 | 1.802 | DETECTION_OF_ABIOTIC_STIMULUS |
| 1.870 | 1.802 | -0.720 | 1.725 | DETECTION_OF_STIMULUS_INVOLVED_IN_SENSORY_PERCEPTION |
| 1.576 | 1.753 | -0.730 | 1.556 | HORMONE_SECRETION |
| 1.695 | 1.504 | -1.197 | 1.495 | SKELETAL_DEVELOPMENT |
| 1.867 | 1.718 | 1.092 | 1.274 | MESODERM_DEVELOPMENT |
| 0.875 | 1.736 | 0.825 | 1.504 | REGULATION_OF_HEART_CONTRACTION |
| 0.909 | 1.959 | -1.410 | 1.177 | GENERATION_OF_A_SIGNAL_INVOLVED_IN_CELL_CELL_SIGNALING |
