# Supplementary material for: Hair follicle epidermal stem cells define a niche for tactile sensation
Source: eLife. 2018 Oct 25;7:e38883. doi: 10.7554/eLife.38883 (PMC6226291; doi:10.7554/eLife.38883)
Supplement: Figure 2—source data 2. [file elife-38883-fig2-data2.docx]

**Figure 2–source data 2. List of ECM proteins screened for upper-bulge localization with immunohistochemical analysis**

| **Gene symbol** | **Protein name (Abbreviation)** | **Antibody ID** | **Manufacturer** | **Upper-bulge localization** | **Tissue localization** |
| --- | --- | --- | --- | --- | --- |
| Aspn | Asporin | ab58741 | abcam | Yes | Upper bulge sensory neuron |
| Crispld1 | Cysteine-rich secretory protein LCCL domain containing 1 (CRISPLD1) | ab123039 | abcam | Yes | Basement membrane zone of upper-bulge |
| Epdr1 | Ependymin related protein 1 (EPDR1) | PA283664 | Cusabio | No | Uncertain |
| Col5a2 | Collagen, type V, alpha 2 (COL5A2) | ab7046 | abcam | Yes | Broadly distributed in the epidermal-dermal basement membrane zone |
| Vwa2 | von Willebrand factor A domain-containing protein 2 (VWA2) | ab111164 | abcam | No | Follicle-arrector pili muscle junction |
| Fam101b | Refilin B | orb183474 | biorbyt | No | Uncertain |
| Ltbp1 | Latent TGF-beta-binding protein 1 (LTBP1) | PA807018 | Cusabio | No | Uncertain |
| Postn | Periostin | ab14041 | abcam | Yes | Basement membrane zone of upper-and mid-bulge |
| Spon2 | Spondin-2 | PA006509 | Cusabio | No | Uncertain |
| Egfl6 | Epidermal growth factor-like protein 6 (EGFL6) | CUK1203 | Fujiwara lab (in house) | Yes | Upper-bulge collar matrix |
| Igfbp5 | Insulin-like growth factor binding protein 5 (IGFBP5) | AF578 | R&D | Yes | Upper bulge sensory neuron |
| Col8a2 | Collagen, type VIII, alpha 2 (COL8A2) | 34099 | USBiological | No | Uncertain |
| Igfbp7 | Insulin-like growth factor binding protein 7 (IGFBP7) | ab129302 | abcam | No | Uncertain |
| Adamtsl4 | ADAMTS-like 4 (ADAMTSL4) |  |  | Not tested |  |
| Col4a3 | Collagen, type IV, alpha 3 (COL4A3) | H31 | Shigei Med. Res. Inst. | Yes | Basement membrane zone of upper-bulge |
| Col4a4 | Collagen, type IV, alpha 4 (COL4A4) | RH42 | Shigei Med. Res. Inst. | Yes | Basement membrane zone of upper-bulge |
